# Feasibility of Using Angioscopy to Visualize the Internal Vessel Wall of the Internal Carotid Artery

**DOI:** 10.1007/s00062-025-01587-4

**Published:** 2025-11-11

**Authors:** Roland Schwab, Nike Zschenderlein, Axel Boese, Stefan Klebingat, Daniel Behme

**Affiliations:** 1 University Clinic for Neuroradiology, University Hospital Magdeburg, Magdeburg, Germany; 2 Research Campus STIMULATE, Otto-von-Guericke University Magdeburg, Magdeburg, Germany; 3 INKA Innovation Laboratory for Image Guided Therapy, Otto-von-Guericke University Magdeburg, Magdeburg, Germany

**Keywords:** Angioscopy, Endovascular, Imaging, Surface, Stroke, Aneurysm

## Abstract

**Background:**

Digital subtraction angiography lacks the ability to visualise intimal surface changes. Angioscopy, in contrast, enables direct imaging of the endoluminal surface, revealing luminal alterations, as established in cardiovascular imaging. Its application in the internal carotid artery (ICA) could allow assessment of pathological changes and the visualization of implant structures such as stents or flow diverters, including their apposition to the vessel wall. This study evaluates the feasibility of using a thin fibre-optic endoscope for angioscopy in the ICA anatomy.

**Methods:**

Five 3D DSA image sets of the ICA with varying anatomies were selected. Tube-based vessel models were fabricated to replicate the corresponding vascular segments, incorporating a measurement scale and, optionally, a stent or a flow diverter. The advancement of a fibre-optic endoscope and the related influence on vessel deformation were assessed in each model. Spatial and colour resolution were evaluated by applying coloured markings to the inner tube surface to simulate vessel wall discolouration. The visibility of structural details, including flow diverter meshes and stent struts, was assessed based on image contrast and sharpness within the angioscope’s defined field of view.

**Results:**

The endoscope was successfully advanced to the distal end in all models. Increased vascular curvature required greater mechanical force for navigation and positioning, leading to observable straightening of the vessel model. In all cases, endoscopic imaging produced clear visualizations of vessel wall markings and implant structures. Stent struts and flow diverter meshes were distinguishable, as were discolorations simulating pathological changes.

**Conclusion:**

Angioscopy of the ICA is technically feasible and enables endoluminal visualization, including implant structures. Feasibility was strongly dependent on vessel curvature and required substantial increases in delivery force in highly tortuous anatomies.

**Supplementary Information:**

The online version of this article (10.1007/s00062-025-01587-4) contains supplementary material, which is available to authorized users.

## Introduction

Intracranial vascular diseases such as aneurysms and atherosclerotic stenoses carry a high risk of stroke or hemorrhage and could be managed with endovascular treatments. These minimally invasive therapies rely on digital subtraction angiography (DSA) and fluroscopy for intraprocedural guidance. Despite enabling indirect visualization of the intimal surface and device wall apposition through three-dimensional (3D) DSA and flat-detector computed tomography angiography, the diagnostic value remains limited due to constraints in contrast resolution and radiopacity, lacking the necessary detail to assess subtle vessel wall pathology or the fine aspects of device apposition and the healing process [1]. In coronary interventions, this limitation has been addressed by employing intravascular imaging techniques. Predominantly, intravascular ultrasound (IVUS) and optical coherence tomography (OCT) can overcome the shortcomings of angiograms. Both modalities demonstrating improved clinical outcomes and being supported by Level 1A evidence [2]. Coronary angioscopy, despite decades of availability, has not yet become standard of care for percutaneous coronary intervention. This success has spurred interest in applying advanced intravascular imaging to neurovascular therapy, where tortuous cerebral anatomy and smaller vessel calibers pose additional challenges [1]. Indeed, a neurovascular OCT system is currently undergoing approval with the flexibility to navigate tortuous cerebral vessels, and early clinical case series have demonstrated that intravascular OCT can identify otherwise occult carotid lesions in stroke patients, and guiding successful intervention [1, 3]. Both IVUS and OCT, however, provide indirect reconstructions of the arterial wall rather than a direct view [4, 5]. Angioscopy, in contrast, involves a fibre-optic endoscope that directly visualizes the intraluminal surface in real time. By producing a true-colour endoscopic image from within the artery, angioscopy can reveal intravascular details, such as wall irregularities, thrombus, or stent structures that may be undetectable on radiographic angiography. Previous coronary angioscopy studies have shown the value of direct optical imaging, for example in identifying vulnerable plaques by their colour and detecting thrombus or incomplete endothelial coverage of stents, information unattainable by angiography alone [6]. Despite its potential, angioscopy has not yet been used routinely in neurointerventions, largely due to technical hurdles like endoscope diameter and stiffness, making it challenging to navigate the demanding cerebral vasculature [7–9]. Recently, a new small diameter and flexible fibre-optic probe was developed that may open the door to a new imaging paradigm in neuroendovascular therapy [10]. The present study investigates the trackability of a micro-angioscope in different internal carotid artery (ICA) anatomies, the effect on vessel deformation by the endoscope, and the corresponding image quality.

## Methods

### Templates

Angiographic datasets acquired between January 2022 and December 2023 were retrospectively reviewed. From this cohort, five ICA cases exhibiting a varying range of anatomical configurations were selected for model reconstruction and testing. Extracranial and cavernous ICA tortuosity were classified into four distinct types. In the extracranial segment, classification was based on the degree of vascular curvature and categorized as straight, tortuous, coiled, or kinked, according to established criteria [11]. Cavernous ICA tortuosity was assessed by measuring the angle between the proximal and distal vessel segments. This angle decreases progressively from Type I to Type IV, with Type IV representing the highest degree of tortuosity [11, 12]. According to the applied classification, the templates were categorized as follows: A—Tortuous, Type I; B—Coiled, Type II; C—Tortuous, Type I; D—Tortuous, Type I; E—Coiled, Type III. A detailed overview is provided in Supplementary Table 1. To build anatomical models, 3D DSA images were processed using the MERCIA Tool 3.1 (Processed using MeVisLab, MeVis Medical Solutions AG, Germany), enabling segmentation and reconstruction of 3D vessel geometries. The Vascular Modelling Toolkit (VMTK, Orobix srl, Italy) was used to extract vascular centrelines, accurately capturing the spatial trajectory and tortuosity of each artery. To allow an optical assessment of model deformation in our experiments, the 3D tortuosity of the centrelines was translated into 2D curvature representations. This was accomplished by calculating the angles between consecutive vectors and projecting them into two dimensions, maintaining the corresponding vector lengths. When the normal vectors of the original centreline exhibited a tilt of 90° or more relative to each other, the planar curve was considered to undergo a directional change, and the sign of the corresponding angles in the two-dimensional representation was inverted. Colour-coding along the centreline was used to visualize curvature orientation and highlight spatial transformations. The resulting 2D representations were algorithmically converted into planar templates, which served as the structural basis for subsequent model construction and analysis. An example is shown in Supplementary Fig. 1.

### Model Construction

Five artificial models (designation A–E) of vascular specimens were created using transparent, kink-resistant polyurethane tubing (Novoplast Schlauchtechnik GmbH, Germany). These tubes provided a homogeneous wall thickness and came with known material properties, allowing for a later analysis of deformation behaviour. The tubing diameter was chosen based on the inner vessel diameter measurements using two methods: manual measurements with Syngo.via (Siemens Healthineers AG, Germany) at five predefined segments of the ICA (pars cervicalis, pars petrosa, pars cavernosa, pars cerebralis and carotid-T), and automated determination of the minimum vessel radius using VMTK (Orobix srl, Italy). Measured vessel diameters ranged between 2.3 and 6.5 mm. A tube with an inner diameter of 2.9 mm is expected to simulate a clinically challenging environment appropriately. In order to reproduce physiological vessel curvature, the tubing was shaped along the 2D templates using a metal mandrin and subsequently heat-fixed.

### Test Setup

**Fig. 1:**
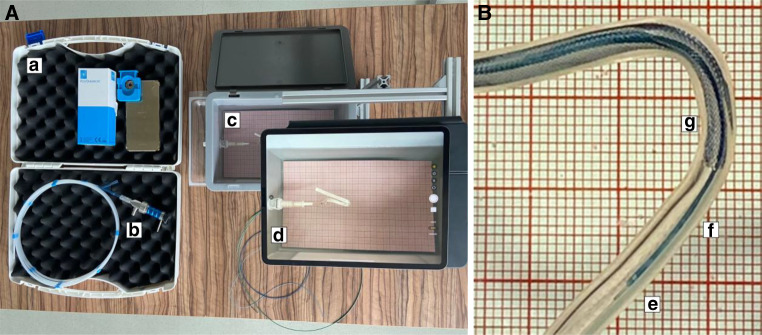
Experimental setup (**A**) including (a) Recording device with the mounting system for endoscopic image capture, (b) Endoscope system with the eyepiece, (c) Enclosure containing the vascular model, and (d) Camera used for documenting the experimental procedure and deformation. Material arrangement within exemplary vascular model (**B**): fiber endoscope (e) (9000 pixels, 0.48 mm diameter, 120° lens; PolyDiagnost, Germany), microcatheter (f) (Prowler Plus 2.8/2.3 F, Cerenovus, USA), Flow Diverter (g) (DERIVO 2 heal Embolisation Device, 5 × 30 mm, Acandis GmbH, Germany)

The models were sequentially positioned in a water-filled plastic container, and the test materials were introduced into the models via a quick-release coupling. The experiments were performed in polyurethane tube models flushed with a water/glycerin solution [13]. To measure model deformations, a millimeter grid was applied to the bottom of the container. A camera was used to record the deformation of the vessel models during instrument insertion, allowing for the estimation of the forces applied to the vessel wall [14]. The material arrangement within the vascular models was as follows: intermediate catheter (5F SOFIA, Microvention, USA), microcatheter (Prowler Plus 2.8/2.3 F, Cerenovus, USA), and a fiber endoscope with 9000 pixels, 0.48 mm (1.5 F) diameter (PolyDiagnost, Germany). In general, all fibers are arranged in a hexagonal pattern to enable a space-saving design. The field of view across the circumference is enabled by forward oriented lenses (0° direction) comparable to a telescope. The lens provides an opening angle of 120° leading to a fisheye effect. The real time imaging and a gradual pull back of the endoscope allows inspection of longer vessel segments. The endoscope is connected to a standard light source and video recording system. Single fibers serve as the light conduit. The flushing protocol was applied as previously described [13]. The experimental setup is shown in Fig. 1.

The intermediate catheter was positioned at the distal end of the model using a guidewire (0.014″ Synchro Select Standard, Stryker, USA), which was removed after successful placement, allowing for subsequent insertion of the microcatheter and endoscope. The applicability of the 5F SOFIA intermediate catheter (Microvention, USA) was assessed in part one of the experiment using the five different vascular models A–E. The catheter was advanced along the microwire (0.014″ Synchro Select Standard, Stryker, USA) with a material testing machine (zwickiLine Z 2.5, ZwickRoell, Germany) at a constant speed of 5 mm/s, while insertion force and distance were continuously recorded. In part two of the experiment, the additional manual advancement of the fiber endoscope through a microcatheter to the distal end of the vascular models was evaluated for positioning, trackability and deformation under complex anatomical conditions. In part three, the endoscopic imaging was evaluated. After distal positioning of the endoscope, the intermediate catheter was repositioned at the proximal end of the model. The endoscope was then retracted to capture the inner wall, including colour-coded markings that were previously applied to the external surface of the tube models. All tests were repeated ten times per model. In model A and D, vascular implants were placed and assessed via imaging. The testing was performed by an interventional neuroradiologist with over 8 years of experience.

### Performance Evaluation

**Fig. 2:**
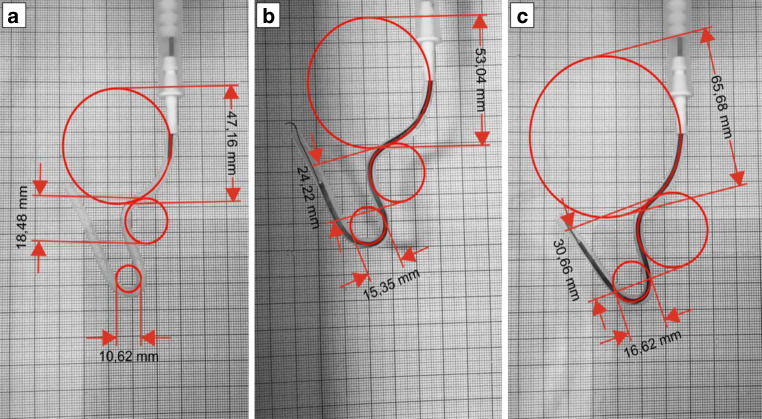
Influence of Instruments on vessel deformation. **a** vessel model in original state, **b** vessel model with inserted catheter and guide wire, **c** vessel model with inserted catheter and angioscope. The stiffness of the instruments leads to a straightening of the vessel model

The trackability of the fibre endoscope was assessed by navigating it through the intermediate and microcatheter systems to the distal segment of each vascular model. This process intends to simulate anatomical constraints and assess navigational precision. Reproducibility was subsequently assessed by the ability to repeatedly position the catheter at the distal end of the vascular model while applying a reasonable level of force. In this context, the required insertion force ranged from 0.61 to 1.4 N. Any deformations of the models during the insertion process, which may arise from catheter manipulation or the application of excessive forces, are systematically documented as shown in Fig. 2; [14].

Endoscopic images are evaluated based on the visibility of colour-coded markings applied to the external surface of the tube models, and of deployed endovascular implants (stent: ACCLINO heal, 4.5. × 20 mm, Acandis GmbH, Germany; flow diverter: DERIVO 2 heal Emolisation Device, 5 × 30 mm, Acandis GmbH, Germany) in two separate vascular models, both classified as “tortuous” and Type I according to the respective tortuosity grading scales [11, 12]. The accessibility of the visualized structures was evaluated using a five-point Likert scale ranging from “very high” to “very low.” This assessment referred to the direct visibility of the aforementioned features in the angioscopic images, rather than to a comparative evaluation with another imaging modality. A “very high” rating indicated complete and unambiguous visibility of all relevant structural or colour features, while “high” referred to slight limitations without significant impact on interpretation. “Medium” denoted moderate visibility with partially discernible features and limited interpretability. A “low” rating reflected substantial visibility reduction, allowing only uncertain or limited assessment. “Very low” indicated that no meaningful evaluation was possible due to a lack of identifiable structures. The ratings were performed by three independent raters (each with more than six years of experience in neurointervention). All raters independently reviewed the recorded angioscopic videos and scored the respective sections. In cases of disagreement, the lower rating was selected.

### Calculation of the Critical Force

The calculations are based on the parameters shown in Supplementary Table 2, with measured values of a, b, and w(a) for the different curvatures listed in Supplementary Table 3.

The elastic bending line w(a) is given by

1 $$w\left(a\right)=\frac{F_{z}*a^{2}*b^{2}}{3*E*I_{y}*l}$$

Rearranging Eq. 1 for Fz yields

1′ $$F_{z}=\frac{w\left(a\right)*\left(3*E*I_{y}*l\right)}{\left(a^{2}*b^{2}\right)}$$

Calculation of the area moment of inertia I for the tubing model, with

2 $$\begin{array}{l} r=1.45*10^{-3}\,m\\ R=2.0*10^{-3}\,m\\ \begin{aligned} I{}&=\frac{\pi }{4}\left(R^{4}-r^{4}\right)\\ &=\frac{\pi }{4}\left(\left(2.0*10^{-3}m\right)^{4}-\left(1.45*10^{-3}m\right)^{4}\right)\\ &=9.09*10^{-12}\,m^{4} \end{aligned}\end{array}$$

Calculation of Fz, considering the modulus of elasticity for all vessel models:

$$E=2{,}5MPa=2{,}5*10^{6}Pa$$

The values for I, E, w(a), a, and b are substituted into Eq. 1′ to obtain F_z_.

Finally, the critical force F_K_ is determined by

3 $$F_{K}=\frac{F_{z}}{2}$$

The calculated values of F_K_ for both catheter and endoscope, together with their percentage deviations, are summarized in Supplementary Table 4.

## Results

### Performance Evaluation

**Fig. 3:**
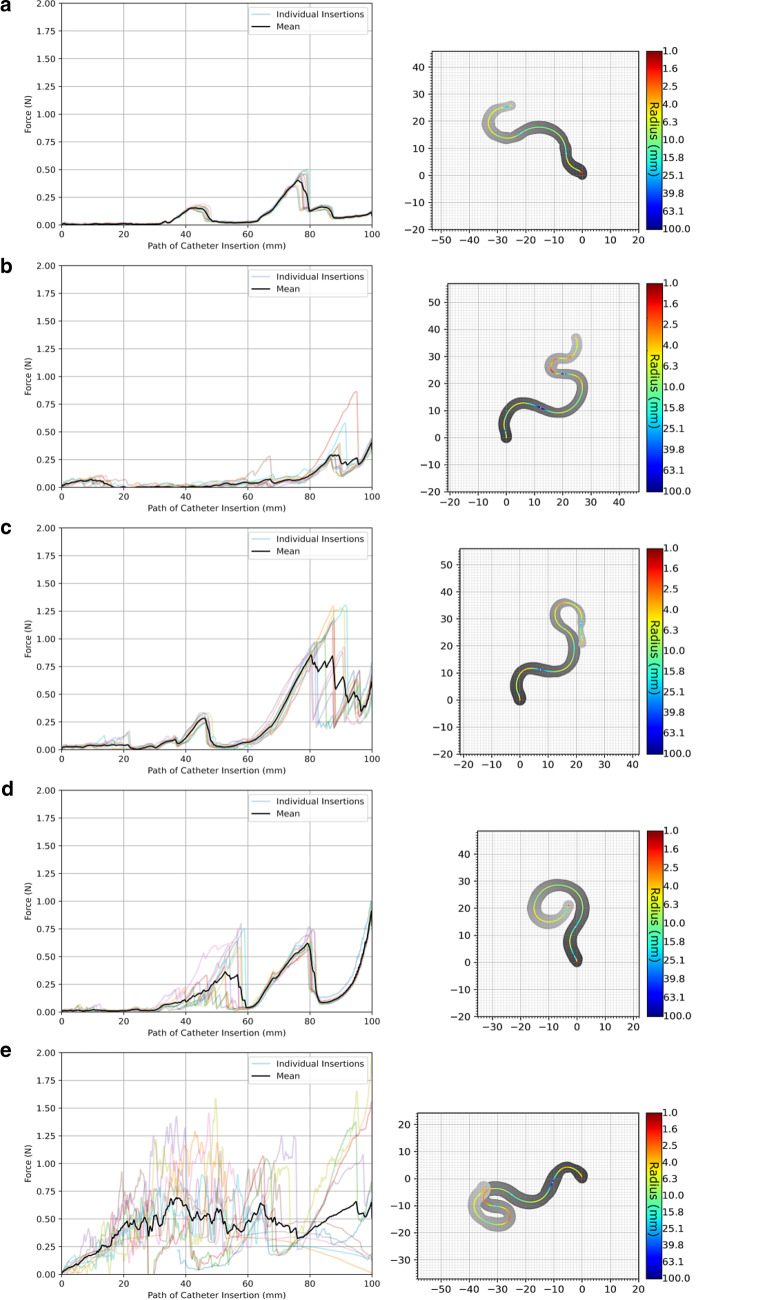
Path-Force-Diagram (left) of individual catheter insertion (colored) including mean values (black) and vessel path with indicated radius (right) for vascular models A‑E

The endoscope was successfully inserted into all vascular models up to the distal end (carotid terminus). In most cases, reproducibility was achieved, as demonstrated for three of the five anatomies (A, B and D). In contrast, two of the anatomies (C and E) exhibited significant limitations in catheter advancement, with the latter in particular showing instances where catheter progression was severely impeded or even impossible. This was primarily due to the increased vascular curvature in these models, coming with smaller radii and greater central angles. As a result, greater force is required to navigate the vascular path and achieve precise endoscope placement. Figure 3 provides an overview of the Path-Force-Diagrams.

Deformation of the vessel models occurs during the process of advancing the catheter system and the endoscope due to the stiffness of the fibres. The radius of curvature increases between 1 and 44% in catheter experiments and between 6 and 68% in endoscope experiments. On average, endoscope application resulted in a 27% higher relative increase in radius of curvature compared to catheter placement. The exact values for each model and curve are summarized in Supplementary Table 5.

### Force Analysis and Friction Effects

The forces exerted on the vessel model wall during endoscope application were calculated using a strength analysis that considers the elastic modulus of the tubing material and changes in vessel curvature. Peak force values are particularly evident in highly curved segments, which can be attributed to straightening induced by the stiffness of the endoscopic system. The exerted forces during catheter and endoscope placements range from 0.03 to 1.51 N and from 0.15 to 6.19 N, respectively. A force increase of 25% to 71% was observed during endoscope application compared to catheter placement under conditions of moderate curvature. This deviation represents the relative increase in force during endoscope use with respect to the force values measured during catheter placement.

### Image Quality and Implant Visibility

**Fig. 4:**
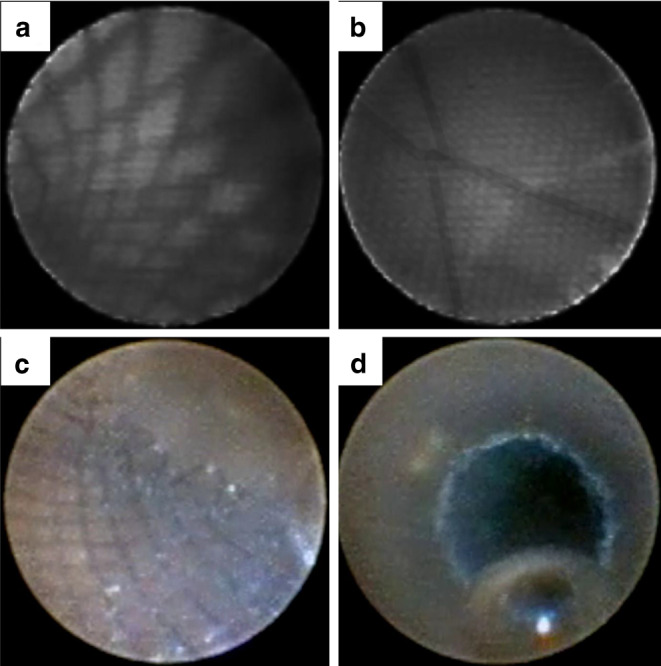
Post-processed endoscopic detail images of a vascular model showing **a** flow diverter braids and **b** stent struts. **c** Unprocessed endoscopic image of the flow diverter at the transition zone of its distal end. **d** Circumferential view of the flow diverter at the proximal end

Angioscopy demonstrates that regardless of the degree of curvature, the vessel lumen can be visualized in model sections within a field of view of 3.0 to 4.5 mm from a distance of 0.84 mm to the 120° lens. Fine structures, such as vessel wall contours, flow diverter meshes, and colour differences, can be visualized with a high degree of clarity. However, the even finer structure of stent struts is only captured to a limited extent, which can be attributed to the image resolution of the endoscope system and the insufficient contrast to the model wall. The incorporation of coloured markings on the exterior surfaces of the model increases the ambient contrast and thereby facilitates a better distinction between the stent struts and the model. Post-processing of the recorded images was essential to achieve clear and reliable visualization. In particular, contrast enhancement was consistently applied to all recordings. Nevertheless, the precise depiction of the stent structure remained inadequate. The visual assessment is shown in Supplementary Table 6 and 7. To improve the visibility of individual components, additional manual adjustments, including selective darkening, were performed. Exemplary endoscpic images are shown in Fig. 4.

## Discussion

Our study demonstrates that intracranial angioscopic imaging is technically achievable and provides high-detail visualization of vascular pathology and implants. Using anatomically realistic ICA models, we were able to track the angioscope through various vessel geometries, including tortuous anatomies. Tracking was generally feasible, though increasing vessel curvature led to higher resistance and friction, requiring greater force to advance the catheter and endoscope. Force measurements showed that in moderate curvatures (radii > 6 mm), advancement remained smooth with push forces under 1.2 N. In contrast, extreme curvatures required forces exceeding 5–6 N. The stiffness of the endoscope in these tight bends sometimes caused straightening of the vessel model, indicating high wall contact forces. In configurations mimicking highly coiled anatomy (models C and E), navigation became unreliable. These observations align with clinical reports that tortuous ICA anatomy complicates endovascular procedures [15]. In previous evaluations of distal access catheters, push forces of 1–2 N have been described as safe, whereas higher values may increase the risk of vessel injury, particularly dissection [16, 17]. In our study, navigation of the angioscope through models with up to moderate curvature can therefore be regarded as safe. By contrast, in models with extreme curvature, push forces exceeded this threshold and were accompanied by vessel straightening. This observation corresponded to the tactile feedback of the performing neurointerventionalist. While navigation under low forces was perceived as smooth and effortless, resistance became critical in extreme curvatures. Once straightening of the vessel model occurred, the performing neurointerventionalist indicated that further advancement would not be attempted under clinical conditions. Although these impressions are subjective and cannot be generalized, they provide an indication that, based on established tactile experience, navigation of the angioscope in vessels with up to moderate curvature appears clinically applicable. Our results mirror in vivo findings, such as those by Srinivasan et al. and Lazaro et al., who successfully navigated a 0.5 mm micro-angioscope in porcine and human cadaveric cerebral vessels [18, 19]. The angioscope’s field of view (3–4 mm at < 1 mm working distance) enabled detailed inspection of surface features such as color variations, stent struts, and flow diverter meshes. These visual details, including metallic sheen and early endothelial coverage, are not visible on X‑ray or ultrasound but have been observed in high-resolution angioscopy studies [18]. Compared to IVUS and OCT, angioscopy provides true-color surface visualization, enabling direct differentiation of thrombus and plaque features. IVUS penetrates deeper but with lower resolution, while OCT offers high resolution without true color. Unlike these cross-sectional modalities, angioscopy delivers a single-perspective view focused on the luminal surface [20–24]. There’s also potential for multimodal approaches, e.g., using narrow-band illumination to improve tissue contrast or fusing angioscopy with OCT to co-register video and cross-sectional data. Such hybrid imaging approaches are already explored [24]. All three methods require catheter navigation through tortuous anatomy. Our angioscopic system uses a 0.5 mm fibrescope, similar in size to a 1.5 F microcatheter, and could be steered through ICA models without significant lumen obstruction. A challenge for angioscopy and OCT is that blood obscures the optical view. In our transparent fluid setup, continuous imaging was possible. In vivo, transient blood clearance by saline flush or balloon occlusion is needed [13, 23]. OCT often uses contrast flush during its 2–3 s acquisitions. Angioscopy may require longer or repeated flushes during the longer real time acquisition, raising safety concerns in cerebral vessels, where ischemia risk is higher [25]. Emerging technologies like scanning fibre endoscopes (SFE) may mitigate this. SFE combine high frame rates, resolution, and small diameters, enabling rapid inspections. One reported use of a prototype SFE in neurointerventions allowed dynamic visualization during device deployment, potentially improving precision beyond what IVUS or OCT can offer in real time [26]. Our study also highlights trade-offs in angioscope design. Image resolution in fibre-optic angioscopy depends on fibre count. More fibres yield sharper images but reduce flexibility. Our 9000-fibre (0.5 mm) angioscope with hexagonal arranged fibres strikes a balance between image clarity and trackability. Larger bundles (e.g., 30,000 fibres at 1.5 mm) provide superior detail in bench tests but were too stiff for sharp curves, echoing limitations reported in prior microendoscope development studies [10]. Looking ahead, neurovascular angioscopes could integrate advanced features like coherent fibre bundles or scanning micro-mirrors to enhance quality while maintaining small diameters and advanced trackabillity. Clinically, direct endoluminal inspection offers several benefits. First, angioscopy could help diagnose intracranial atherosclerosis, particularly in patients with cryptogenic strokes, by revealing high-risk plaque features. Second, angioscopy could guide intra-procedural decisions. For example, after deploying a flow diverting stent, real-time endoscopic imaging could confirm device expansion and wall apposition or detect branch coverage. Information that conventional angiography only infers [26]. In cases of thrombosis or dissection, angioscopy could potentially rapidly identify the issue and guide intervention. Coronary studies have shown its ability to detect microthrombi or dissection flaps invisible on angiograms, suggesting similar utility in neurovascular procedures [24]. Third, angioscopy could be valuable for follow-ups. In our study, imaging of a flow diverter clearly showed its struts and wall contact. In vivo, such visualization could assess healing, e.g., endothelial coverage or hyperplasia on implanted devices. Animal studies have already shown that follow-up angioscopy can detect partial endothelial coverage of flow diverting stents, providing visual confirmation where angiography cannot [18]. This could guide clinical decisions about discontinuing dual antiplatelet therapy [26]. In summary, our findings establish the feasibility of neurovascular angioscopy, identify key technical challenges in extreme tortuous anatomy, and outline how this modality could complement existing intravascular imaging. As technology advances, angioscopy may evolve into a valuable tool for diagnosis, intervention guidance, and post-treatment assessment in neurovascular care.

## Limitations

We acknowledge that our study has limitations. Primarily, the experiments were performed in polyurethane tube models with water/glycerin solution, not in real arteries with blood flow. While the geometric realism is high, factors like pulsatile motion, blood opacity, and endothelial layer were not present. Thus, the ease of angioscope navigation and the crystal-clear imagery in our tests represent a best-case scenario. In a real intracranial artery, careful management of blood clearance will be necessary [25]. Neurovascular angioscopy also raises safety considerations. Even a brief interruption of cerebral blood flow or manipulation within a fragile artery can risk ischemia or vessel injury. Reassuringly, preliminary animal work reported no vessel damage from angioscopy usage in the carotid territory [23], and the catheter sizes are similar to existing clinical devices. Another limitation is that our angioscope was a forward-viewing fiber endoscope without tip steering or deflection. In tortuous or branching anatomy, some areas of the wall might be difficult to angle towards. Side-viewing optics or rotatable probe designs could address this in future prototypes. Finally, the study was an initial feasibility assessment with qualitative outcomes. Our findings require confirmation in further in vivo studies involving real patients. Future research should quantify the imaging resolution in microns, the area of wall seen per image, and the minimum detectable lesion size. Comparative studies with IVUS and OCT in the same vessel would also be valuable to objectively map out the strengths of angioscopy.

## Conclusion

In summary, our in vitro study demonstrates that neurovascular angioscopy in the internal carotid artery is technically achievable and can provide clear imaging of the inner lumen and endovascular devices, but feasibility is strongly dependent on vessel curvature and may require critical increases in delivery force in extremely tortuous anatomies. Despite the challenges, the ability to directly visualize the inner vessel wall and endovascular implants offers a new dimension of information for neurointerventional procedures. Angioscopy could evolve into a useful adjunct for complex cerebrovascular interventions, e.g. guiding flow diverter placement, checking coiled aneurysm sacs for residual leaks, or differentiating plaques from clots during thrombectomy. Continued technological improvements, informed by both cardiology experience and neuro-specific requirements, will be key to translating angioscopic imaging from the laboratory into clinical practice. With further development, cerebrovascular angioscopy has the potential to become a valuable tool for enhancing the safety and efficacy of interventions, ultimately improving outcomes for patients with stroke and aneurysmal disease.

## Supplementary Information

The supplementary section provides detailed tables summarizing the experimental procedures and corresponding results. Furthermore, it contains additional figures depicting the individual 3D model reconstructions.
