# Supplementary material for: Feasibility of Using Angioscopy to Visualize the Internal Vessel Wall of the Internal Carotid Artery
Source: Clin Neuroradiol. 2025 Nov 11;36(2):359–67. doi: 10.1007/s00062-025-01587-4 (PMC13319176; doi:10.1007/s00062-025-01587-4)
Supplement: Supplementary file 1 — The supplementary section provides detailed tables summarizing the experimental procedures and corresponding results. Furthermore, it contains additional figures depicting the individual 3D model reconstructions. [file 62_2025_1587_MOESM1_ESM.pdf]

Online supplemental data

Supplementary Figure 1 Illustration of an internal carotid artery curvature derivation. Vessel course with black centerline and corresponding 3D polygonal curve representing tortuosity (A), and (B) corresponding 2D template with highlighted vessel's radius of curvature and indication of angioscope insertion direction (black arrow).

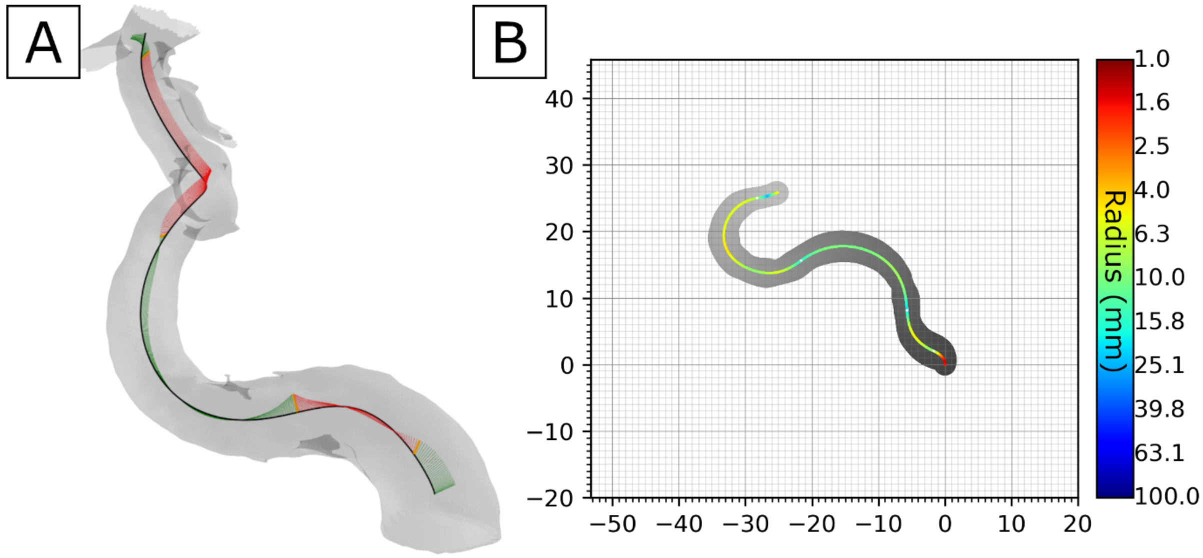

Supplementary Table 1 Classification of Anatomical Characteristics and ICA Tortuosity in the Five Vascular Models

Vascular Model	Anatomical Characteristics	Extracranial ICA Tortuosity	Cavernous ICA Tortuosity
A	Elongated vessel course, short pars cerebralis, relatively large curvature radius	Tortous	Typ I
B	Small curvature radius in the petrous and cavernous segments	Coiled	Typ II
C	Cervical segment extends particularly far proximally, long pars cerebralis, relatively large curvature radius	Tortous	Typ I
D	Representative of the majority of the sample, no notable anatomical deviations	Tortous	Typ I

E	Small curvature radius in the petrous and cavernous segments	Coiled	Typ III

Supplementary Table 2 Parameters and units used in the subsequent calculations.

**Symbol**	**Unit**	**Designation**	**Remark**
a, b	m	Distance	
l	m	Distance	l = a + b
r, R	m	Radius	
E	N/m^2	Modulus of elasticity	E = σ/ϵ
F_z	N	Force	1 N = 1 kg $*$ m/s^2
I	m^4	Area moment of inertia	I = $\int z^2 \, d\, A$

Supplementary Table 3 Measured values of *a*, *b*, and *w(a)* for the different curvatures C1 – C4 under catheter and endoscope insertion.

		**w(a) (10^{-3}m)**		**Distance a (10^{-3}m)**		**Distance b (10^{-3}m)**	
**Model**	**Curvature**	Catheter	Endoscope	Catheter	Endoscope	Catheter	Endoscope
**A**	**C1**	2	7	11	17	10	13
	**C2**	3	4	12	13	10	8
**B**	**C1**	1	5	14	16	14	10
	**C2**	5	13	6	10	7	4
	**C3**	5	15	9	10	6	10
	**C4**	6	17	7	10	6	7
**C**	**C1**	0,5	4	5	11	10	8
	**C2**	4	6	8	8	9	9
	**C3**	7	7	10	7	8	6
**D**	**C1**	1	1	13	14	7	2
	**C2**	3	5	7	8	12	10
**E**	**C1**	3	8	12	10	15	8
	**C2**	3	8	11	3	8	7
	**C3**	4	7	9	5	7	10
	**C4**	5	11	11	7	12	12

Supplementary Table 4 Measured values of F_K (N) for catheter and endoscope, including percentage deviation.

		F_K (N)		Deviation (%)
**Model**	**Curvature**	Catheter	Endoscope	
**A**	**C1**	0,12	0,15	25
	**C2**	0,16	0,27	69
**B**	**C1**	0,03	0,18	500
	**C2**	1,26	3,88	208
	**C3**	0,88	1,03	17
	**C4**	1,51	2,01	33
**C**	**C1**	0,10	0,34	240
	**C2**	0,45	0,67	49
	**C3**	0,67	1,76	163
**D**	**C1**	0,08	0,67	738
	**C2**	0,28	0,48	71
**E**	**C1**	0,09	0,77	756
	**C2**	0,25	6,19	2376
	**C3**	0,55	1,43	160
	**C4**	0,23	1,01	339

Supplementary Table 5 Radius of curvature for curves C1–C3 of the a) vessel model, b) with catheter, and c) with catheter and endoscope, including percentage deviation from a).

Vascular Model A												
	Radius C1 (mm)			Radius C2 (mm)			Radius C3 (mm)					
	a)	b)	c)	a)	b)	c)	a)	b)	c)			
	23,58	26,52	32,48	9,24	12,11	15,33	5,31	7,67	8,31			
Deviation (%)		12	38		31	66		44	57			
Vascular Model B												
	Radius C1 (mm)			Radius C2 (mm)			Radius C3 (mm)			Radius C4 (mm)		
	a)	b)	c)	a)	b)	c)	a)	b)	c)	a)	b)	c)
	14,80	15,97	21,27	8,44	8,91	12,69	6,03	6,93	9,33	9,39	10,26	10,57
Deviation (%)		8	44		6	50		15	55		9	13
Vascular Model C												

	Radius C1 (mm)			Radius C2 (mm)			Radius C3 (mm)					
	a)	b)	c)	a)	b)	c)	a)	b)	c)			
	8,63	9,73	12,06	4,79	5,58	7,74	5,14	5,19	8,63			
Deviation (%)		13	40		16	62		1	68			
Vascular Model D												
	Radius C1 (mm)			Radius C2 (mm)								
	a)	b)	c)	a)	b)	c)						
	9,19	9,31	11,00	4,78	5,57	6,75						
Deviation (%)		1	20		17	41						
Vascular Model E												
	Radius C1 (mm)			Radius C2 (mm)			Radius C3 (mm)			Radius C4 (mm)		
	a)	b)	c)	a)	b)	c)	a)	b)	c)	a)	b)	c)
	4,99	5,36	5,30	7,62	9,22	10,64	4,99	5,55	5,64	4,38	4,40	5,13
Deviation (%)		7	6		21	40		11	13		1	17

Supplementary Table 6 Likert Scale Assessing the Visibility of Individual Stent Struts or Flow Diverter Meshes

Device setup	Very High	High	Medium	Low	Very Low
Flow Diverter	x				
Flow Diverter + Colour Markings		x			
Stent			x		
Stent + Colour Markings			x		

Supplementary Table 7 Likert Scale Assessing the Visibility of Colour Differences

Device setup	Very High	High	Medium	Low	Very Low
Flow Diverter + Colour Markings		x			
Stent + Colour Markings		x			
